# Manatees in Zoological Parks throughout the World: History, State, and Welfare

**DOI:** 10.3390/ani13203228

**Published:** 2023-10-16

**Authors:** Yann Henaut, Fabienne Delfour

**Affiliations:** 1 Laboratorio de Conducta Animal, Grupo Académico Interacción, Adaptación y Biodiversidad, El Colegio de la Frontera Sur (ECOSUR), Chetumal 77014, Quintana Roo, Mexico; 2 Animaux et Compagnies, 31000 Toulouse, France; fabienne_delfour@yahoo.com

**Keywords:** manatee, welfare, zoos, ecology, behaviour, cognition, management

## Abstract

**Simple Summary:**

Fewer than 200 manatees are hosted permanently by zoological parks worldwide. Their living conditions differ considerably from zoo to zoo. Recent research on ecology, acoustics, cognition and behaviour has changed the perception humans have on manatees with implications for their welfare. Using the five-model approach, this study examines our current knowledge on manatees’ behaviour and cognition, and suggests ways to improve their welfare in zoological parks.

**Abstract:**

The order Sirenia comprises several species of manatees and one species of dugong. These popular marine mammals are relatively recent acquisitions to zoological parks throughout the world. As far as we know, there are less than 200 manatees, mostly American, a few African, and ever less Amazonian, currently in zoological parks. American manatees are predominantly found in zoos in Europe, North America, and in some Asian countries, while African ones are present exclusively in Asian zoos. The living conditions of captive manatees differ considerably from zoo to zoo (i.e., numbers, sex ratio, outdoor vs. indoor habitats, complex vs. simple habitats). Most research on manatee behaviour has been relatively recent, and studies on cognition, sociality, and ecology have a significant impact on our perception of manatee needs and management, with wider implications for their welfare. In the wild, manatees demonstrated various cognitive capacities; spatial memory and learning abilities play an important role in their daily life in a complex and dynamic environment. Furthermore, there is evidence that these mammals are more social animals than expected. Individuals show various personality traits on the boldness–shyness continuum and their sociality varies. All those parameters are important in terms of animal welfare. Several behavioural studies showed that standardized enrichment programs benefit and ensure the welfare of captive zoo animals. However, obtaining accurate information on the presence of manatees in zoos, living conditions, management, and consequently welfare remains challenging. This study examines the current knowledge on manatee behaviour and cognition and then discusses different approaches to improving the welfare of this charismatic marine mammal in zoological parks.

## 1. Introduction

Over recent years, manatees have become more and more popular. In the past, very little was known about these species as they were rarely observed, and ignorance was only nourished by myths about sirenians (i.e., mermaids). In Florida, for example, there was widespread aversion towards them, with claims that they consumed all the fish or attempted to bite swimmers, besides being generally regarded as a hindrance to boating activities [1]. Thankfully there has been a recent shift in human perception of these animals. Manatees are now protected, and there are conservation and education campaigns aimed at the general community. Nowadays, people perceive sirenians in a positive way, particularly the American species, *Trichechus manatus* [1]. Manatees also became attractive aquatic mammals for visitors to zoos. The study of animal behaviour is key for the development of management policies for the conservation of rare or endangered species [2,3]. Studying manatee behaviour in the wild is difficult due to their elusive nature and turbid waters; however, zoos can provide opportunities to study the behaviour of captive individuals and therefore play a crucial role in manatee conservation. Consequently, the welfare of captive manatees and their conservation in the wild are intimately linked [4,5].

Sirenians are aquatic mammals that include three species of manatee (*Trichechidae*) and one species of dugong (*Dugongidae*). They are aquatic herbivorous mammals living in estuaries, swamps, rivers, marine wetlands, and coastal waters. The sirenian order is closely related to *Elephantidae* and is part of the *Afrotheria* clade; this superorder includes many endangered mammals [6]. The dugong (*Dugong dugon*, Muller 1776) is the last living species of its genera and has a wide distribution over 40 countries throughout the Indo-West Pacific region [7]. Dugongs present several obvious differences from manatees [6,8]. In this paper, we focus on the three species of manatees.

The African manatee, *Trichechus senegalensis* (Link 1795), is an endangered species [9] with around 10,000 mature individuals according to the International Union for Conservation of Nature (IUCN) (https://www.iucnredlist.org/species/22104/97168578 (accessed on 4 July 2023)). Their distribution range includes the western and central coasts of Africa. Little is known about this African species, but scientific research is underway, which is vital for its conservation (see for example [10]). The remaining two species, the Amazonian manatee *T. inunguis* (Natterer 1883) and the West Indian manatee *T. manatus* (Linnaeus 1758), are found within the Americas. The former inhabits the Amazonian basin and is particularly vulnerable due to habitat fragmentation [11]. The population of this particularly cryptic species is difficult to estimate but according to the IUCN varies between 8000 and 30,000 individuals (https://www.iucnredlist.org/species/22102/43793736 (accessed on 4 July 2023)). The West Indian manatee is distributed from the Florida coast to Brazil and comprises two subspecies: *T. m. latirostris*, the Florida manatee, is present on the Florida coast and west along the Gulf of Mexico coast to Texas. The distribution range of the Antillean manatee, *T. m. manatus*, stretches from the Gulf and Caribbean coast of Mexico to the coast of north-eastern Brazil, but also around the islands of the Lesser Antilles [12]. These two subspecies are classified as vulnerable by the IUCN [13] which estimates a population of approximately 4100 and 3300 individuals for the Antillean and Florida manatee, respectively (https://www.iucnredlist.org/species/22103/9356917 (accessed on 4 July 2023)). A recent survey carried out by the Florida Fish and Wildlife Conservation Commission estimates a considerably larger figure of 5733 for the Florida subspecies (https://myfwc.com/research/manatee/research/population-monitoring/synoptic-surveys/ (accessed on 4 July 2023)). However, population numbers can fluctuate due to the difficulty of observing manatees in their natural habitats.

Currently, manatees are exhibited in several zoological parks around the world but information about their numbers, species, subspecies, and current location is often difficult to obtain. This information is essential to gain an insight into the animals’ living conditions, including their habitat design and social grouping, and to devise future actions to support institutions in promoting their welfare and effective management practices.

As previously mentioned, human perception of manatees and their behaviours have evolved over time. Initially, they were considered very passive animals but according to recent studies they appear more social with higher cognitive abilities than previously thought [14]. A biocentric approach that considers their ecological and social context [14] and research on their cognition in the wild [15] are key components needed to help zoos manage manatee groups and to contribute to their conservation. Reep and Bauer [16] report numerous anecdotes on Florida manatee behaviour that reveal that their intelligence is an important factor that requires further research. Furthermore, the presence of manatees in zoological institutions worldwide and an increase in tourism activities in their natural habitat highlight the need to consider manatee cognition to provide insights and perspectives on the welfare of these animals, whether under human care or interacting with humans in their natural habitats.

Animal welfare is an important component of conservation concerns [17]. Zoos and aquariums are important partners in scientific and conservation programs and should be more involved in animal welfare and wildlife conservation actions. For the care of manatees, zoos can rely on a wealth of veterinary scientific literature and on the EAZA best practice guidelines (https://www.eaza.net/assets/Uploads/CCC/2018-Antillean-Manatee-EAZA-Best-Practice-Guidelines-Approved.pdf, accessed on 4 July 2023) to ensure best practice in their management (see [18]. However, manatee welfare remains poorly studied and documented with a lack of knowledge on their behaviour and cognition [14] and concerns have recently been raised for sirenian welfare in both the wild and under human care in captivity; these were discussed recently, for example, see [18,19]. These issues need to be addressed through cognitive biocentric paradigms.

Aim of the paper. We will discuss the welfare of manatees under human care by focusing on four main points: (1) Manatees under human care and enrichment, (2) Manatee cognition (learning ability, personality, interactions), (3) Manatee behaviour and sociality, and (4) Consequences and perspectives for manatee welfare, conservation, and rehabilitation.

## 2. Manatees under Human Care and Enrichment

Information on manatees in zoological and other institutions is difficult to obtain and there are very few studies on captive manatees in the scientific literature. We have therefore sought information from zoological parks and the Internet and remain cautious about the results. We have only considered zoos that host these animals permanently and where manatees are kept solely for the purpose of being exhibited to the public. We have not considered rehabilitation centres where manatees are temporarily kept under human care before being released. Information was gathered by reading scientific papers and books [18,20,21] and by visiting Internet websites (https://species360.org/ (accessed on 4 July 2023), https://www.zooborns.com (accessed on 4 July 2023), https://zooinstitutes.com (accessed on 4 July 2023), https://www.zoochat.com (accessed on 4 July 2023), https://www.zootierliste.de (accessed on 4 July 2023)). We also talked directly to veterinarians working in zoos to have the list of manatees officially present in zoos in Europe and America. The director of the veterinary department at Beauval Zoo (France) sent us a list of manatees present in zoological parks and information on their management. This list covered all European zoological parks; however, not all Asian zoos and only some of the institutions on the American continent were included. We also obtained the list of manatees held in zoos in Mexico, thanks to information shared by a manager working at Dolphin Discovery (Mexico), and in America. We also used Species360 ZIMS (Zoological Information Management, ZIMS Release 2.3—25 January 2016) to complete the information.

Worldwide, information on manatees in zoos is very scattered and sometimes incomplete (e.g., distinction between subspecies) and not organized internationally. This study highlights the need for zoos to organize themselves to effectively share information on manatees. Sharing information between zoological parks around the world could be of great benefit for the management of these animals, for research and conservation, and for improving practices linked to their welfare. For example, there is very little published information on the African species and its state in Asian zoos; therefore, behavioural and cognitive research on this and other species of manatee are vital if we are to gain an understanding of their specific behavioural and ecological requirements for conservation and welfare.

**Figure 1:**
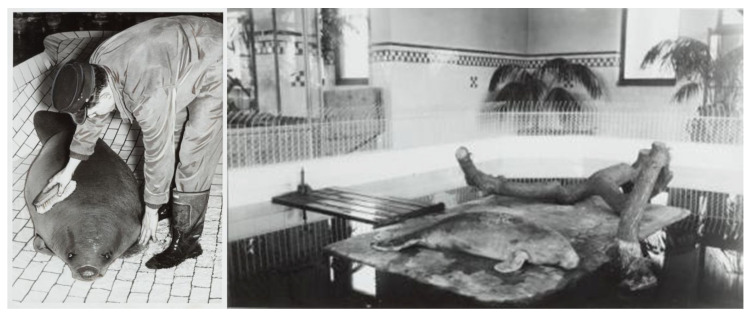
African manatee Antwerp, Belgium (unknown date: tentatively between 1922–1923, 1929 or between 1954 and 1973 found in https://www.zoochat.com/community/media/african-manatee-or-lamantin.119545/ (accessed on 4 July 2023).

Two *T. senegalensis* individuals captured in the Congo River (Africa) were displayed at the beginning of the 20th century in Antwerpen Zoo (Belgium) (Figure 1) [22]. The first individual survived around one year in Antwerpen Zoo between 7 October 1922 to 19 March 1923; the second manatee was introduced to the zoo in 1929 and only survived a few months. The last West African manatee in Antwerpen arrived in 1954 and lived there alone for 16 years [22]. African manatees were also reported living in zoos in Rotterdam and in the Netherlands. Asian countries (mainly China, Japan, Taiwan, and the Republic of Korea) imported several African manatees between 1975 and 2013 (*n* = 44 according to CITES database) from Cameroon, Guinea, Guinea Bissau, and Ivory Coast, until the species was assigned to the CITES annex I [22]. African manatees are reported, for example, in Safari World (Bangkok, Thailand), Hangzhou Zoo, and Dalian Laohutan Ocean Park in China, COEX Aquarium Seoul (South Korea) and the Toba Aquarium (Japan). This list of Asian countries hosting *T. senegalensis* remains open and constantly changes in relation to translocations between institutions, births, and mortality of animals. It is important to note that East Asian countries are the only ones that host this endangered African species and therefore have an important role to play regarding its welfare, management, research, and conservation.

The Amazonian manatee, *T. inunguis*, is reported in several rescue and education centres, such as in Peru (https://rarec.org (accessed on 4 July 2023)), where a calf that will be released shortly was rescued in January 2023. This manatee species is reported as permanent in two zoos. One single individual male called “Junto” is living in Atagawa Tropical and Alligator Garden (Japan); another individual male called “Tapajós” may be observed in the São Paulo aquarium (Brazil). However, in the past, Amazonian manatees were displayed in zoos in Germany (*n* = 4 zoos), Netherlands (*n* = 2 zoos), Poland (*n* = 1 zoo), and the United Kingdom (*n* = 1 zoo).

Walsh and Blyde [18] mention the case of the American manatee, *T. manatus*, a species that was translocated to European or North American zoos in the 19th century but did not survive the journey or endured only a few months in captivity. The short lifespan of manatees was attributed to the limited knowledge and veterinary information available; however, the emergence of advanced techniques during the second half of the 20th century has significantly improved their chances of survival and increased their life expectancy. Currently, reproductive success is relatively high. Based on the information we obtained, *T. manatus* is present in at least 27 zoological institutions (Table 1) spanning 11 countries with a total of 155 manatees reported, although this number is an estimation. The total number of animals in zoos and other institutions fluctuates over time due to ongoing births and deaths and partly because we were unable to obtain the exact number of zoological parks that host *T. manatus*. We did not consider orphan manatees under human care, such as those at wildlife rescue centers that are kept in captivity prior to release, for example, the young male manatee “Pompeyo” in Quintana Roo, Mexico [23].

American manatees are present in several zoos, predominantly in Europe (10 zoos in seven countries) and North America (*n* = 15, mostly in Florida and seven in Mexico, principally in Quintana Roo). In North America, manatees are generally in zoos located close to their natural range of distribution. At least two zoos host American manatees in Asia. The Antillean subspecies is not always mentioned, so in Table 1 we have presented the results by combining all *T. manatus* individuals. However, it is reasonable to assume that the subspecies present in the USA, principally in Florida, is *T. m. latirostris* with a total of 44 individuals and that *T. m. manatus* (*n* = 111) is present in Mexican (*n* = 47), Asiatic (*n* = 23), and European zoos (*n* = 40); one female called “Ayurami” is the only Antillean manatee in the USA, hosted in the Dallas World Aquarium. We have no reliable information on which subspecies of manatee is predominant in European zoos. The determination of the subspecies may be important since management and reproductive programs in zoos may change according to ecological and behavioural differences between the Caribbean and the Florida manatees. Although the two subspecies appear similar, their natural environments differ, and genetic and morphological differences are well established [14]. These variations can lead to differences in behaviour and needs in captivity, making it imperative that zoos tailor their management practices according to the subspecies.

**Figure 2:**
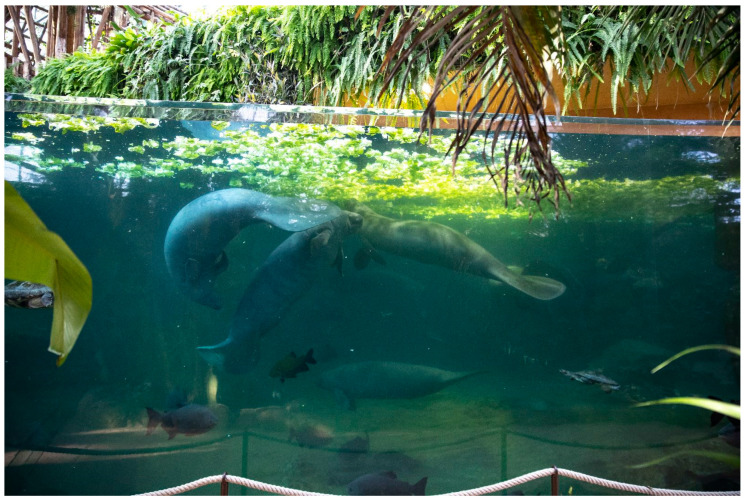
Antillean manatees in Beauval Zooparc equatorial dome (Credit photo Zooparc de Beauval).

Artificial manatee habitats vary considerably among zoos. Some zoos keep manatees in equatorial domes (i.e., indoor settings) where the animals live in a complex environment with other aquatic species, surrounded by terrestrial animals (i.e., birds, monkeys, insects). For example, in Beauval Zooparc (France), manatees live in tanks with the Arau turtle (*Podocnemis expensa)*, arapaima (*Arapaima gigas*), and tambaqui (*Colossoma macropomum*) (Figure 2). However, even if this environment might be attractive for visitors since it mimics manatees’ natural habitats, we do not know how the manatees perceive their environment and how it impacts their welfare. Under human care, manatees also live in outdoor pens built in natural water areas. In this case, manatees are surrounded by other species that sometimes visit or observe them through a fence, and they are exposed to natural sounds and anthropogenic activities (Figure 2). However, in other institutions, whether indoors or outdoors, manatees live in artificial habitats with concrete walls. Does this impact the welfare of manatees? This is not known as there is no available study on their welfare.

Artificial or semi-artificial habitats are limited in terms of space available when we consider the natural home range of manatees; these aquatic mammals travel large distances for resources and reproduction or to avoid adverse weather conditions [14]. Is the opportunity to swim long distances essential for manatee welfare? Again, there is a lack of research and information on this component of manatee behaviour. In zoological settings, manatees are also exposed to anthropogenic noises (e.g., water pump, ambient background music, or visitors). Research conducted on wild populations reveals that noise may affect manatees, leading them to avoid noise-polluted areas [24] and interactions with humans [25]. In Florida, an increase in tourism focused on manatees has led to a rise in harassment towards them [26], while in Mexico, boat trips to observe manatees are known to disturb these animals, leading them to swim away from the noise created by the boats, humans, or even drones [27]. Manatees are a tourist attraction in their natural areas of distribution, particularly in resort areas such as Florida and the Mexican Caribbean; however, such economic activities come with an ecological cost as anthropogenic activities have a detrimental effect on the welfare of these charismatic creatures [1]. On the other hand, we have no information on whether noise or visitors has a negative impact on the welfare of manatees in zoos.

The social environment for captive manatees varies from zoo to zoo, with some individuals living in large groups of males, females, and calves and others limited to a pair of individuals. Manatee social behaviour appears to be adaptable, with individuals displaying both solitary and group behaviours at different times. Social groups may contain many individual manatees [14]. Manatee sexual behaviour is limited and mainly for reproductive purposes; they do not exhibit the same level of sociality as in some primates or cetaceans [28]. Do manatees need to live in large groups, and with males and females together? Ortega-Argueta and Castelblanco-Martínez [20] suggest that animals that have spent a long time in captivity or are born in captivity become attached to their carertakers; they are docile animals and are more easily exposed to danger and in fine less able to survive in the wild. However, there is no evidence that differences in habitats, interactions, and social environment negatively impact manatee welfare.

Flint and Bonde [19] suggest applying the five freedoms approach for manatees living in artificial conditions: freedom (1) from hunger and thirst, (2) from discomfort, (3) from disease, pain, injury, (4) to express their natural behaviour observed in the wild, and (5) from mental suffering. These standards help prevent decision making based on anthropomorphism rather than on scientific objective facts on the biological and behavioural needs of a given animal. However, the application of the five freedoms depends on our knowledge of manatee ecology, behavioural ecology, and cognition (i.e., learning capacities, social cognition, and personality). To improve the efficacy of this method, we propose adopting a biocentric approach [14].

Furthermore, considering the ANSES (French Agency for Food, Environmental, and Occupational Health and Safety, 2018) definition of animal welfare is: “An ongoing positive physical and mental state resulting from the satisfaction of the animal’s behavioural and physiological needs and expectations. This state varies according to the perception of the situation by the animal” (ANSES, 2018, n° 2016-SA-0288), animal welfare assessment must include resource-based and animal-based indicators, following the five domains approach developed [29] (i.e., nutrition, environment, health, behaviour, and mental states). This approach is implemented in the Dolphin WET (Welfare Evaluation Tool) developed by the welfare committee of the European Association of Aquatic Mammals (www.eaam.org) and the multispecies Ackonc-AWA tool [30]. An urgent need exists for the development of an objective assessment grid to assess the welfare of manatees, based on extensive and appropriate research.

Contemporary zoos aim to enhance the welfare of animals by providing mental and physical stimulation through management practices involving environmental enrichment and training programs. There are five enrichment categories regularly provided to zoo animals: (1) social (i.e., conspecifics, other animals, people (caretakers and visitors), other (e.g., mirror), (2) cognitive (i.e., mental stimulations, novel experiences), (3) physical/habitat (e.g., climbing/perching structures, substrates, dens, refuges, temperature), (4) sensory (e.g., tactile, olfactory, auditory, visual), and (5) food (e.g., novel food items and presentations).

Environmental enrichment programs should increase the display of species-appropriate behaviour through the addition of various stimuli [31]. The goal is to increase positive behaviours (e.g., exploration, affiliative behaviours, play), to enhance behavioural diversity, to provide animals the opportunities to make choices and a degree of control over their environment, see [31]. Several zoos already use the SPIDER model [32] which includes six steps for designing successful programs: setting goals, planning, implementing, documenting, evaluating, and readjusting. Zoo animal welfare assessments should include indicators targeting enrichment provided by caretakers. Training programs could also provide animals with cognitive enrichment and physical exercises that may increase the individuals’ welfare [31].

Currently, there is no research available on enhancing the welfare of captive manatees through enrichment programs. For such programs to be effective, it is imperative that the animals’ behaviours, cognitive abilities, and sensory capacities are considered.

## 3. Manatee Cognition (Learning Abilities, Personality, Interactions)

Numerous anecdotes and observations indicate that manatees possess significant cognitive abilities—they display play behaviours, interact with their physical and social environment (i.e., conspecifics and other species including humans), mimic others’ behaviours, and demonstrate social learning abilities [16]. For example, when human-reared manatees are reintroduced to their natural environment, they exhibit less severe responses, specifically avoidance, to anthropogenic disturbances than their wild conspecifics [33]. However, habituation of animals to people is potentially problematic as it may result in harmful encounters with them; manatees would benefit more from avoiding humans [20]. This example highlights the behavioural plasticity of manatees in changing environments and the fact that they can adapt and accommodate to human care when living in captivity.

There is little experimental research on the learning abilities of manatees, although evidence suggests that they may share cognitive abilities like those of their closest relatives, elephants [34], which show a remarkable aptitude for learning and memory [14,15,16]. In a recent study, Henaut et al. [35] show that a manatee was able to discriminate and associate geometrical forms using food rewards. Surprisingly, its scores remained high a year later, even though the animal was not subject to reinforcement.

Object manipulations and play with objects were observed in manatees during different scientific experiments [35,36] and were interpreted as play behaviours. This play activity was also observed during interactions with conspecifics or with other species including humans [16]. However, manatee temperament [36,37] may modify social interactions, play behaviours, and manipulations of social or physical objects. While some manatee individuals have been acknowledged for their curiosity and cognitive prowess, it is possible that certain individuals are more social, inquisitive, and bolder and could benefit more from social enrichment than their shier and less interactive counterparts [36]. It is essential that these factors are considered when assessing manatee welfare. A shy manatee will tend to interact less with enrichment devices provided and will show more neophobic behaviours than a bold conspecific, for instance.

A recent study showed that captive Antillean manatee calves display anticipatory vocal behaviours before regular feeding events [38]. Anticipatory behaviour can be defined as the animals’ ability to use cues from the surroundings to foresee what is about to happen [39]; it has been thought to be a potential welfare indicator in bottlenose dolphins [40,41]. We suggest monitoring anticipatory behaviours in captive manatees as part of welfare assessments.

Manatees enjoy manipulating objects and possess excellent learning capabilities; therefore, to promote exploratory and inquisitive behaviours, the provision of appropriate stimuli is essential. Furthermore, given that activities for manatees in zoos typically follow a daily routine, we strongly recommend monitoring their anticipatory behaviour to gain insight into their welfare.

## 4. Manatee Behaviour and Sociality

Sociality in manatees is not easy to interpret and appears to be flexible. There are limited data available on this aspect for African and Amazonian species [42]. The two subspecies of *T. manatus* differ in their living habits (e.g., large groups for Florida manatees versus small groups for Antillean manatees) and, as observed in captivity, appear to be tolerant of each other (no aggression) and of younger animals [43]. In terms of social management and welfare, we cannot conclude that manatees should be kept in groups, nor can we specify their ideal composition. However, we propose that group composition may significantly affect manatee welfare and should therefore be studied as a potential welfare indicator.

Living in a group provides opportunities for social animals to develop and display their vocal communication abilities [44] and may increase social play behaviours and affiliative behaviours that are potential positive welfare indicators [45]. Therefore, it is essential that manatees in zoos have the opportunity to display positive social interactions with conspecifics and/or humans.

Sensorial and occupational enrichment should also be provided. Manatees possess a well-developed sense of touch (tactile sense) and demonstrate interest in a great variety of objects they spend time with and enjoy manipulating [16]. In their natural habitats, individuals are frequently observed engaging in tactile exploration or interaction with their surroundings, as well as exhibiting harassment towards other species such as alligators [16]. During experimental research, some individuals approached and interacted with new objects [35,36]. This approach and interaction behaviour, or neophilia, is largely determined by temperament. Individuals who are extroverted are more likely to engage with newly introduced objects in their surroundings as opposed to individuals who are introverted [36]. As observed by Henaut et al. [14], when new objects are introduced to a pool, manatees interacted with the objects but also increased their moving activities without displaying stereotypical circling behaviour [35]. Similarly, when one manatee associated food with various plastic geometrical shapes that were introduced to its pool, there was a noticeable increase in general activity, moving, and interactions with the shape [35]. The provision of objects and the association of an object with food appear to work as an effective occupational strategy to improve manatee activity and could then potentially increase *in fine* their welfare. Significantly, when introducing objects, particularly underwater objects, manatee vocalizations increase, and individuals tend to interact more [46]. It is, however, difficult to determine whether these behaviours are positive or negative, for example, symptomatic of a stress caused by a change in their habitat. Introduced objects seem to have a positive impact on extroverted manatees; however, it remains uncertain whether they have a favourable, neutral, or adverse impact on introverted individuals. Considering this, we recommend conducting additional research on the introduction of novel objects and its correlation with food as a way to increase manatee engagement in zoological settings.

Manatees are sensitive to sounds; they can associate the sounds of trucks or some motorboats with unpleasant experiences and consequently swim away [16]. Wild manatees avoid areas with human activities, while manatees that were raised by humans display neutral behaviour [33]. Vocalizations are essential for intraspecific communication and new research describes how manatees interact using vocalizations, e.g., [38,44]. As observed in other species [47], noise pollution may not only induce stress in animals but also affect their communication. However, it is uncertain how noises (e.g., water pumps, music, or visitors) affect manatees in captivity. Noise pollution is a recognized stressor for numerous species, particularly aquatic mammals [48,49,50,51]. Further research is required to improve our understanding of this human-caused stressor when assessing manatee welfare in zoological institutions. It is important to establish whether manatee response to new noises is negative, neutral, or positive and further research is needed on how temperament modulates manatee responses in general, for example, when encountering new objects [36]. Research into acoustic aspects and their relationship with manatee welfare is crucial for establishing effective management strategies in zoos that aim to prevent stressful situations and modify the use of sounds according to manatee personality.

Bills et al. [52] report that male manatees use chemoreception to detect females in oestrus by identifying odours/chemicals produced by their anal glands. Barboza and Larkin [53] established that taste is used by manatees to detect toxic food, saltwater, and freshwater. Chemoreception may assist manatees in the detection of conspecifics, resources such as food and water, and their palatability; therefore, careful consideration should be given to the quality of their food and water. Manatee enrichment programs should incorporate a wider variety of feeding rations, types of food, and feeding behaviours. However, there are few studies on chemoreception in manatees and more scientific research is necessary to achieve a better understanding of this sense.

Vision in manatees is dichromatic; they distinguish blue and green from greys and discriminate brightness [54,55]. Several accounts suggest that manatees use underwater and aerial vision [16]. Manatees can also associate visual cues with food rewards and to recall this connection after one year [35]. Visual stimuli could be enriching or provoke stress; therefore, their relationship with manatee welfare also merits further study.

Manatees are close relatives of elephants, see, for example, [14], and many observations suggest that they possess remarkable memory and learning abilities [16], as is the case with their pachyderm cousins. Although these observations lack experimental and controlled scientific contexts, they provide substantial evidence to support this claim [14]. They are also regarded as being highly trainable and possessing a strong aptitude for learning as evidenced by several observations conducted by veterinarians [14]. Manatees travel large distances searching for scattered resources in a constantly changing environment, suggesting that they require good memory and learning abilities to successfully find food, water, and conspecifics, see [14]. Further experimental research is required to gain an understanding of the role cognition plays in manatee behaviour. One potential approach to improving the welfare of manatees in human care could be cognitive enrichment. Cognitive enrichment has shown promising potential in dolphins [56,57,58] and elephants [59]. We highly recommend conducting research that considers the role of manatee personality and affective states while assessing the advantages of enrichment programs on the animals’ welfare.

## 5. Conclusions

Today, the five domains approach is widely used for evaluating the welfare of animals, including those in zoos. In this context, it is crucial that researchers and zoo curators develop a tool based on the five domains approach that can be implemented specifically to assess the welfare of manatees in zoos and rehabilitation centres. This tool will be species-specific and will rely on experts’ knowledge and scientific literature. However, in most cases, zoo animals will remain in an anthropogenic environment, while those kept in rehabilitation or species conservation facilities will eventually be released in the wild: their needs differ and thus their management should fulfil different requirements. In the latter case, it is essential that human contact be minimized to prevent habituation and the captive environment should closely resemble the animal’s natural habitat. However, we believe that several parameters such as environmental richness hold true for both manatees in zoos and those that will be released from captivity into the wild, such as for conservation purposes. Enrichment has been demonstrated to provide animals under human care with behavioural, cognitive, and health benefits across several species. In this study, we demonstrated that social (e.g., social group) and sensory enrichments, including chemoreceptive, visual, and tactile stimuli, in addition to monitoring anticipatory behaviours and manatee personality and affective states, have the potential to improve the welfare of manatees in captivity.

## 6. Future Directions

Despite the lack of information on manatee ecology and behaviour, numerous observations and some recent studies have provided us with information to start assessing their welfare, enrich their physical and social environment, and finally to improve their rehabilitation and conservation (Table 2). We acknowledge the paucity of scientific knowledge on manatee cognition, perception, learning capacities, sensitivity, and emotions. In addition, we note that most studies on manatee behaviour are anecdotal and very few are experimental (Table 2). In this regard, and given the situation of manatees in zoos and the need to ensure their welfare and conservation, the ethology and cognition of manatees must be seriously considered and studied in the near future. We should be grateful to zoos that allow researchers to observe and study these animals with the aim of improving their welfare and contributing to their conservation.

## Figures and Tables

**Table 1:** List of zoos and parks hosting American manatees in 2023. The number of animals may vary over time.

Continent	Country	Zoological Park	N
Europe	Denmark	ODENSE, Odense Zoologiske Have	2
	Denmark	RANDERS, Randers Regnskov	2
	France	BEAUVAL, Zoo Parc de Beauval	15
	France	PARIS ZOO, Parc Zoologique de Paris (MNHN)	3
	Germany	NURNBERG, Tiergarten der Stadt Nürnberg	3
	Germany	DUISBURG, Zoo Duisburg gGmbH	2
	Italy	GENOVA AQ, Acquario di Genova	2
	Netherlands	ARNHEM, Royal Burgers’ Zoo	3
	Poland	WROCLAW, ZOO Wroclaw	6
	Spain	FAUNIA, Parque biológico De Madrid	2
America	USA (Florida)	EPCOT, Living Seas	2
	USA (Florida)	Homosassa Springs Wildlife State Park	6
	USA (Florida)	Jacksonville Zoo and Gardens	2
	USA (Florida)	LOWRY, ZooTampa at Lowry Park	23
	USA (Florida)	Mote Marine Lab and Aquarium	2
	USA (Ohio)	Cincinnati Zoo and botanical garden	3
	USA (Ohio)	Columbus Zoo and Aquarium	6
	USA (Texas)	Dallas World Aquarium	1
	Mexico (Quintana Roo)	Dolphin Discovery (4 centres: Isla Mujeres, Cozumel, Dreams and Puerto Aventuras)	16
	Mexico (Quintana Roo)	XCARET	10
	Mexico (Quintana Roo)	XELHA	2
	Mexico (Jalisco)	Guadalajara Zoo	2
	Mexico (Vera Cruz)	Aquarium del Puerto de Vera Cruz	8
	Mexico (Chiapas)	Aluxes Ecopark	4
	Mexico (Tabasco)	*Yumká* wildlife and safari park	5
Asia	Japan	Okinawa Churaumi Aquarium	4
	Singapore	SINGAPORE, Singapore Zoo	19

**Table 2:** Factors that may affect manatee behavioural responses considered as positive or negative in term of welfare. ?: no data, +: impact, -: no studies published. We distinguish positive and negative aspects, as some factors may affect manatees positively or negatively depending on current manatee temperament, the nature of the object/individual/animal they interact with, whether the manatee is wild or captive, or the animal’s history and experiences. A [16,24,27,33,47]; B [16]; C [25]; D [16,35,36]; E [38].

Factors	Positive Effect	Negative Effect	Reference
Artificial habitat complexity	?	?	-
Size of the area	?	?	-
Anthropogenic noises	?	+ ^(a)^	A
Interaction with other species	+ ^(a)^	+ ^(a)^	B
Interaction with people	?	+ ^(a)^	C
Sociality	?	?	-
Chemoreception	?	?	-
Visual stimuli	+ ^(1)^	?	D
Tactile stimuli	+ ^(2)^	?	D
Emotion and anticipatory behaviours	+ ^(b)^	?	E

^a^ (observed for wild manatees only), ^b^ (observed in rehabilitation centre), ^1^ (attraction and associative learning), ^2^ (object manipulation, learning).

## Data Availability

Not applicable.

## References

[1] Goedeke T.L. (2004). In the Eye of the Beholder: Changing Social Perceptions of the Florida Manatee. Soc. Anim..

[2] Curio E. (1996). Conservation needs ethology. Trends Ecol. Evol..

[3] Marske K.A., Lanier H.C., Siler C.D., Stein L.R. (2023). Integrating biogeography and behavioral ecology to rapidly address biodiversity loss. Proc. Natl. Acad. Sci. USA.

[4] Kleiman D.G. (1992). Behavior research in zoos: Past, present and future. Zoo Biol..

[5] Patrick P.G., Matthews C.E., Ayers D.F., Tunnicliffe S.D. (2007). Conservation and Education: Prominent Themes in Zoo Mission Statements. J. Environ. Educ..

[6] Marsh H., O’Shea T.J., Reynolds J.E. (2011). Ecology and Conservation of the Sirenia, Dugongs and Manatees.

[7] Nishiwaki M., Kasuya T., Miyasaki N., Tobayama T., Takaota T. (1979). Present distribution of the dugong in the world. Sci. Rep. Whales Res. Inst..

[8] Rowlatt U., Marsh H. (1985). The Heart of the Dugong (*Dugong dugon*) and the West Indian Manatee (*Trichechus manatus*) (Sirenia). J. Morphol..

[9] Keith Diagne L. (2015). *Trichechus senegalensis*. The IUCN Red List of Threatened Species.

[10] Factheu C., Rycyk A.M., Kekeunou S., Keith-Diagne L.W., Ramos E.A., Kikuchi M., Takoukam Kamla A. (2023). Acoustic methods improve the detection of the endangered African manatee. Front. Mar. Sci..

[11] Marmontel M., de Souza D., Kendall S. (2016). *Trichechus inunguis*. The IUCN Red List of Threatened Species.

[12] Vianna J.A., Bonde R.K., Caballero S., Giraldo J.P., Lima R.P., Clark A., Marmontel M., Morales-Vela B., De Souza M.J., Parr L. (2006). Phylogeography, phylogeny and hybridization in trichechid sirenians: Implications for manatee conservation. Mol. Ecol..

[13] Deutsch C.J., Self-Sullivan C., Mignucci-Giannoni A. (2008). *Trichechus manatus*. The IUCN Red List of Threatened Species.

[14] Henaut Y., Charles A., Delfour F. (2022). Cognition of the manatee: Past research and future developments. Anim. Cogn..

[15] Bauer G.B., Reep R.L. (2022). Manatee cognition in the wild: An exploration of the manatee mind and behavior through neuroanatomy, psychophysics, and field observations. Anim. Cogn..

[16] Reep R.L., Bauer G.B. (2023). Anecdotal Accounts of Manatee Behavior: Conservation and Management, Behavioral Ecology, and Cognition. Aquat. Mamm..

[17] Maple T.L., Perdue B.M. (2013). Zoo Animal Welfare. Zoo Animal Welfare.

[18] Walsh M.T., Blyde D.J., Butterworth A. (2017). Sirenian Health and Well-Being in Managed Care. Marine Mammal Welfare.

[19] Flint M., Bonde R.K., Butterworth A. (2017). Assessing Welfare of Individual Sirenians in the Wild and in Captivity. Marine Mammal Welfare.

[20] Ortega-Argueta A., Castelblanco-Martínez D.N. (2018). Is captive breeding a priority for manatee conservation in Mexico?. Oryx.

[21] Suzuki A., Ueda K., Segawa T., Suzuki M. (2019). Fecal microbiota of captive Antillean manatee *Trichechus manatus manatus*. FEMS Microbiol. Lett..

[22] Laudisoit A., Collet M., Muyaya B., Mauwa C., Ntadi S., Wendelen W., Guiet A., Helsen P., Baudouin M., Leirs H. (2017). West African Manatee *Trichechus senegalensis* (LINK, 1795) in the Estuary of the Congo River (Democratic Republic of the Congo): Review and Update. J. Biodivers. Endanger. Species.

[23] Castelblanco-Martínez D.N., Sanchez-Okrucky R., Padilla-Saldívar J.A., Niño-Torres C.A., Garcés-Cuartas N., Perez-Flores J.S., Blanco-Parra M.P., Lara-Sánchez L., Julio-Cardoso S., Cruz-Varela L. (2021). Pompeyo: A manatee calf rescued in Laguna Milagros, Quintana Roo, Mexico. Sirenews.

[24] Miksis-Olds J.L., Donaghay P.L., Miller J.H., Tyack P.L., Nystuen J.A. (2007). Noise level correlates with manatee use of foraging habitats. J. Acoust. Soc. Am..

[25] Slone D.H., Butler S.M., Reid J.P., Kleen J., Palmer J. (2023). How do ambient conditions and management actions affect manatee movements and habitat use?. J. Wildl. Manag..

[26] Sorice M.G., Shafer C.S., Ditton R.B. (2006). Managing Endangered Species within the Use–Preservation Paradox: The Florida Manatee (*Trichechus manatus latirostris*) as a Tourism Attraction. Environ. Manag..

[27] Landeo-Yauri S.S., Castelblanco-Martínez D.N., Henaut Y., Arreola Maria R., Ramos E.A. (2021). Behavioural and physiological responses of captive Antillean manatees to small aerial drones. Wildl. Res..

[28] Furuichi T., Connor R., Hashimoto C., Yamagiwa J., Karczmarski L. (2014). Non-conceptive Sexual Interactions in Monkeys, Apes, and Dolphins. Primates and Cetaceans, Primatology Monographs.

[29] Mellor D.J., Beausoleil N.J., Littlewood K.E., McLean A.N., McGreevy P.D., Jones B., Wilkins C. (2020). The 2020 Five Domains Model: Including Human–Animal Interactions in Assessments of Animal Welfare. Animals.

[30] Racciatti D.S., Feld A., Rial L.A., Blanco C., Tallo-Parra O. (2022). Ackonc-AWA: A multi-species animal welfare assessment protocol for wild animals under human care to overcome the use of generic welfare checklists. Front. Vet. Sci..

[31] Lauderdale L.K., Shorter K.A., Zhang D., Gabaldon J., Mellen J.D., Granger D.A., Miller L.J. (2022). Environmental Enrichment Factors Associated with the Activity Level of Bottlenose Dolphins under Professional Care. J. Zool. Bot. Gard..

[32] Alligood C., Leighty K. (2015). Putting the “E” in SPIDER: Evolving trends in the evaluation of environmental enrichment efficacy in zoological settings. Anim. Behav. Cogn..

[33] Campos D.O., Souto A., Telino Júnior W.R., Borges J.C.G., Schiel N., Alves M.D.O. (2023). Behaviour and occurrence of the Antillean manatee (*Trichechus manatus manatus*) in relation to habitat characteristics and the influence of human activities in a protected area in north-eastern Brazil. Aquat. Conserv. Mar. Freshw..

[34] Murata Y., Nikaido M., Sasaki T., Cao Y., Fukumoto Y., Hasegawa M., Okada N. (2003). Afrotherian phylogeny as inferred from complete mitochondrial genomes. Mol. Phylogenetics Evol..

[35] Henaut Y., Lara-Sánchez L.E., Morales-Vela B., Machkour-M’rabet S. (2020). Learning capacities and welfare in an Antillean manatee, *Trichechus manatus manatus*. Comptes Rendus. Biol..

[36] Charles A., Henaut Y., Saint Jalme M., Mulot B., Lecu A., Delfour F. (2022). Studying Antillean manatees’ (*Trichechus manatus manatus*) temperament in zoological parks: Exploration of boldness, sociality and reactivity to humans. Appl. Anim. Behav. Sci..

[37] Lucchini K., do Val H.G.P., Umeed R., de Azevedo C.S., Löffler Niemeyer Attademo F., dos SMelo L.I., de Oliveira Luna F., Bezerra B. (2023). Personality traits in captive Antillean manatees (*Trichechus manatus manatus*) in Brazil and perspectives for the release of individuals. Appl. Anim. Behav. Sci..

[38] Ramos E.A., Brady B., Lasala J.A., Liebschner A., Obbink S., Walker Z., Rebello M., Magnasco M.O. (2023). Antillean manatee calves in captive rehabilitation change vocal behavior in anticipation of feeding. Zoo Biol..

[39] Jensen A.L.M., Delfour F., Carter T. (2013). Anticipatory behavior in captive bottlenose dolphins (*Tursiops truncatus*): A preliminary study. Zoo Biol..

[40] Clegg I.L., Rödel H.G., Boivin X., Delfour F. (2018). Looking forward to interacting with their caretakers: Dolphins’ anticipatory behaviour indicates motivation to participate in specific events. Appl. Anim. Behav. Sci..

[41] Bigiani S., Pilenga C. (2023). Using Anticipatory Behavior to Detect the Change in Interest in an Activity Repeated Several Times and Avoid Habituation in Bottlenose Dolphins (*Tursiops Truncatus*). Appl. Anim. Welf. Sci..

[42] Morales-Vela B., Olivera-Gómez D., Reynolds III J.E., Rathbun G.B. (2000). Distribution and habitat use by manatees (*Trichechus manatus manatus*) in Belize and Chetumal Bay, Mexico. Biol. Conserv..

[43] Henaut Y., Becerra-Lopez S.P., Machkour-M’Rabet S., Morales-Vela B., Winterton P., Delfour F. (2010). Activities and social interactions in captive Antillean manatees in Mexico. Mammalia.

[44] Brady B., Hedwig D., Trygonis V., Gerstein E. (2020). Classification of Florida manatee (*Trichechus manatus latirostris*) vocalizations. J. Acoust. Soc. Am..

[45] Held S.D.E., Špinka M. (2011). Animal play and animal welfare. Anim. Behav..

[46] Charles A., Henaut Y., Saint Jalmes M., Mulot B., Lecu A., Delfour F. (2023). Visual and acoustic exploratory behaviors toward novel stimuli in Antillean manatees (*Trichechus manatus manatus*) under human care. J. Comp. Psychol..

[47] Berkhout B.W., Budria A., Thieltges D.W., Slabbekoorn H. (2023). Anthropogenic noise pollution and wildlife diseases. Trends Parasitol..

[48] Bruck J.N. (2023). A deeper understanding of noise effects on cetaceans. Learn. Behav..

[49] Lemos L.S., Haxel J.H., Olsen A., Burnett J.D., Smith A., Chandler T.E., Torres L.G. (2022). Effects of vessel traffic and ocean noise on gray whale stress hormones. Sci. Rep..

[50] van der Knaap I., Ashe E., Hannay D., Bergman A.G., Nielsen K.A., Lo C.F., Williams R. (2022). Behavioural responses of wild Pacific salmon and herring to boat noise. Mar. Pollut. Bull..

[51] Mills S.C., Beldade R., Henry L., Laverty D., Nedelec S.L., Simpson S.D., Radford A.N. (2020). Hormonal and behavioural effects of motorboat noise on wild coral reef fish. Environ. Pollut..

[52] Bills M.L., Samuelson D.A., Larkin I.V. (2013). Anal glands of the Florida manatee, *Trichechus manatus latirostris*, a potential source or chemosensory signal expression. Mar. Mammal Sci..

[53] Barboza M.L.B., Larkin I.V. (2020). Functional morphology of the taste buds of Florida manatee, *Trichechus manatus latirostris*. Mar. Mammal Sci..

[54] Griebel U., Schmid A. (1996). Color Vision in the Manatee (*Trichechus manatus*). Vis. Res..

[55] Griebel U., Schmid A. (1997). Brightness discrimination ability in the west Indian manatee (*Trichechus manatus*). J. Exp. Biol..

[56] Clark F.E. (2017). Cognitive enrichment and welfare: Current approaches and future directions. Anim. Behav. Cogn..

[57] Matrai E., Ng A.K., Chan M.M., Gendron S.M., Dudzinski K.M. (2020). Testing use of a potential cognitive enrichment device by an Indo-Pacific bottlenose dolphin (*Tursiops aduncus*). Zoo Biol..

[58] Mátrai E., Gendron S.M., Boos M., Pogány Á. (2022). Cognitive Group Testing Promotes Affiliative Behaviors in Dolphins. J. Appl. Anim. Welf. Sci..

[59] French F., Mancini C., Sharp H. (2018). High tech cognitive and acoustic enrichment for captive elephants. J. Neurosci. Methods.

